# Assessment of Nocturnal Blood Pressure: Importance of Determining the Time in Bed—A Pilot Study

**DOI:** 10.3390/jcm13082170

**Published:** 2024-04-09

**Authors:** Niklas Pilz, Viktor Heinz, Gianfranco Parati, Ralph Haberl, Elisabeth Hofmann, Gert Küchler, Andreas Patzak, Tomas L. Bothe

**Affiliations:** 1 Institute of Translational Physiology, Charité—Universitätsmedizin Berlin, Corporate Member of Freie Universität Berlin and Humboldt-Universität zu Berlin, 10117 Berlin, Germany; andreas.patzak@charite.de; 2 Institute of Physiology, Center for Space Medicine and Extreme Environments Berlin, Charité—Universitätsmedizin Berlin, Corporate Member of Freie Universität Berlin and Humboldt-Universität zu Berlin, 10117 Berlin, Germany; viktor.heinz@charite.de (V.H.); tomas-lucca.bothe@charite.de (T.L.B.); 3 Department of Medicine and Surgery, University of Milano-Bicocca, 20126 Milan, Italy; gianfranco.parati@unimib.it; 4 Department of Cardiology, Istituto Auxologico Italiano, Institute for Treatment and Research (I.R.C.C.S.) S. Luca Hospital, 20149 Milan, Italy; 5 Cardiologic Medical Office, 80634 Munich, Germany; ralph.haberl@herzimblick.de; 6 SOMNOmedics GmbH, 97236 Randersacker, Germany; eh@somnomedics.de (E.H.); gk@somnomedics.de (G.K.)

**Keywords:** nocturnal blood pressure, dipping pattern, cardiovascular risk, individualized medicine, time in bed

## Abstract

**Objectives:** Nocturnal blood pressure (BP) monitoring is essential for evaluating cardiovascular risk and guiding treatment decisions. However, the standardized narrow-fixed nighttime period between 10 p.m. and 6 a.m. may not accurately reflect individual sleep schedules. This pilot study aimed to investigate the comparability between the standardized nighttime period and actual time in bed (TIB) regarding BP assessment. Further, our goal was to evaluate the clinical relevance of the observed BP differences. **Methods:** A total of 30 participants underwent 24 h ambulatory blood pressure monitoring (ABPM). Patient-specific TIB was precisely assessed through an accelerometer and a position sensor from the SOMNOtouch NIBP™ (SOMNOmedics GmbH, Randersacker, Germany). We analysed the effect of considering individual TIB as nighttime instead of the conventional narrow-fixed interval on the resulting nocturnal BP levels and dipping patterns. **Results**: We observed differences in both systolic and diastolic BP between the standardized nighttime period and the TIB. Furthermore, a notable percentage of patients (27%) changed their dipping pattern classification as a function of the nighttime definition adopted. We found strong correlations between the start (r = 0.75, *p* < 0.01), as well as the duration (r = −0.42, *p* = 0.02) of TIB and the changes in dipping pattern classification. **Conclusions**: Definition of nocturnal period based on the individual TIB leads to clinically relevant changes of nocturnal BP and dipping pattern classifications. TIB is easily detected using a body position sensor and accelerometer. This approach may thus improve the accuracy of cardiovascular risk evaluation and enhance treatment strategies.

## 1. Introduction

Blood pressure (BP) assessment is a central component of cardiovascular health evaluation, aiding in the diagnosis, management, and prevention of various medical conditions [1].

Daytime BP measurements have traditionally been employed for cardiovascular risk assessment, but nocturnal BP monitoring provides additional valuable information. Nocturnal BP levels and the dipping pattern have proven to be strong predictors of cardiovascular events. They enable the identification of patients at higher risk of cardiovascular diseases, including heart attack, stroke, and renal dysfunction [2,3,4,5,6]. By improving cardiovascular risk profile estimates through the inclusion of a more accurate nocturnal BP assessment, clinicians might implement more focussed interventions and devise personalized treatment approaches [1,7]. 

Ambulatory blood pressure monitoring (ABPM) is the gold-standard method for evaluating nocturnal BP, automatically capturing BP measurements at regular intervals over a 24 h period [8,9]. 

In hypertension diagnostics, the nighttime is usually defined as the standard time period from 10 p.m. to 6 a.m. BP measurements taken during this period are referred to as nocturnal BP levels [7]. Nonetheless, defining the time asleep as the interval between 10 p.m. and 6 a.m. may not always accurately reflect individual sleep patterns. Sleep duration and behaviour vary widely among different patients [10,11]. Unfortunately, directly determining sleep is methodologically challenging and practically unfeasible during ABPM in daily practice [12]. An alternative approach proposed to estimate nocturnal BP is based on the determination of the nighttime period by assessing the time in bed (TIB). The TIB is the time spent in bed with the intention to sleep. TIB is easier to assess than the actual sleep time and more closely reflects the individual sleep period than a fixed time interval [13]. Definition of TIB could be based on patient’s diary, but evidence is available that self-reported sleep times differ strongly from TIB determined from actigraphy [14,15]. It has been shown that actigraphy-derived TIB is the best-suited surrogate measure of true sleep time derived from full polysomnography [14,15].

By examining the comparability between the standardized nighttime period (10 p.m.–6 a.m.) and the TIB, we could gain insights into the accuracy of current nocturnal BP assessment practices and potentially optimize cardiovascular risk evaluation. However, determining the actual time asleep without disturbing the patient’s sleep is a challenging task. Given the limited reliability of patient diaries, an approach relying on a body position sensor in combination with actigraphy to determine the TIB appears to be promising.

Therefore, the primary objective of this study was to investigate to what degree the standardized nighttime period from 10 p.m. to 6 a.m. adequately represents the TIB and to explore the respective impact of the definitions of nighttime based on these two different approaches on nocturnal BP levels assessment. Additionally, we aimed to assess the clinical relevance of the resulting nocturnal BP differences in individual patients.

## 2. Methods

### 2.1. Participants

Patients aged 18 years and older with a clinical indication, such as the diagnosis or treatment monitoring of hypertension for an ABPM, were included in this pilot study, as we were aiming to identify the relevant effects in clinical patients. Inclusion criteria comprised a systolic BP interarm difference of less than 10 mmHg, a diastolic BP interarm difference smaller than 5 mmHg, and an upper arm circumference ranging from 22 to 32 cm. 

Exclusion criteria encompassed patients with an implanted pacemaker or ICD, chronic atrial fibrillation, aortic coarctation, subclavian steal syndrome, peripheral artery disease, or Raynaud syndrome. 

The study was conducted in an outpatient cardiologic medical office in Munich, Germany. Part of the data was recently published in a work considering the artefacts of oscillometric ABPM [16]. We had to exclude one participant from this work who was included in the artefact paper due to a measurement failure of a body position sensor. To maintain a total of 30 subjects, we conducted a measurement on an additional patient. This leads to closely related but not identical datasets of both works [16].

### 2.2. Devices

In this study, we used a BoSo TM-2430 ABPM device (BOSCH + SOHN GmbH u., Co., KG, Jungingen, Germany; outside of Germany: *A&D TM-2430*, A&D Inc., Tokyo, Japan) placed on the subjects’ dominant arm [17]. 

Additionally, we attached a SOMNOtouch NIBP™ (SOMNOmedics GmbH, Randersacker, Germany) to the contralateral arm. The SOMNOtouch NIBP™ incorporates a 3-channel ECG and an SpO_2_-Sensor to determine the pulse arrival time [18,19]. The ECG-electrode located on the left upper thorax includes an accelerometer for the assessment of motor activity and determination of TIB. The devices were synchronized to a precision of one second.

### 2.3. Study Procedure

**Figure 1:**
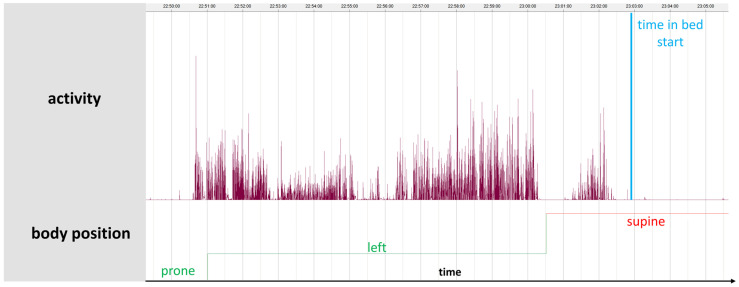
Original data: The upper row illustrates the body activity, while the lower row represents body position. The vertical blue line indicates the start of the time in bed. Accordingly, the activity after this marker is close to zero.

Upon arrival, participants provided written informed consent to participate in the study. The ABPM device was programmed to measure BP at a 15 min interval between 6 a.m. and 10 p.m., and every 30 min between 10 p.m. and 6 a.m., as recommended at the commencement of the study. The measurements were conducted over a period of approximately 24 h to capture the participants’ full circadian BP profile (Figure 1).

### 2.4. Determination of the TIB

Given the limited reliability of patient diaries, individual TIB measurements were determined by recording body position using a chest-worn sensor of the SOMNOtouch NIBP™. The beginning of the TIB was set when there was a change in body position from upright to a lying body position (right, left, prone, or back), with accompanying decrease in motor activity to an average of less than 10 mg (a thousandth of the Earth’s gravitational force) over 3 min. The end of the TIB was set accordingly when there was a change from a lying body position to an upright position, accompanied by an onset of motoric activity of more than 10 mg over a 3 min average.

### 2.5. Statistical Analysis

We conducted all analyses using Python 3.9 and the SciPy statistics library [20]. Descriptive analyses of the dataset composition and the disagreement in the definition of nocturnal BP levels between the TIB and the conventional nighttime definition based on the narrow-fixed time interval (10 p.m.–6 a.m.) were analysed. Between 10 p.m. and 6 a.m., we compared the average BP values of the measurements taken during the TIB with those taken outside of the TIB using paired t-test. Beyond this, we calculated the effect of measurements during TIB on mean nocturnal BP and dipping classification. We used the Wilcoxon test for paired non-parametric samples to compare the individual mean nocturnal BP based on a fixed nighttime interval with that based on TIB. Lastly, we evaluated the correlation between patient individual deviation from the expected 10 p.m.–6 a.m. rest period and changes in dipping pattern. All but the descriptive analyses were tested against an alpha-level of *p* = 0.05.

### 2.6. Ethics and Data Availability

The study was conducted according to the guidelines of the Declaration of Helsinki and received ethical approval from the local ethics committee at Ludwig-Maximilians-Universität München, Munich, Germany (approval number: 19-026, approval date: 11 March 2019). All included individuals gave informed consent to participate in this study. The study dataset is available upon reasonable request sent to the corresponding author.

## 3. Results

### 3.1. Dataset Composition

We included 30 subjects in this pilot study. Their demographic characteristics, including age, height, weight, body mass index (BMI), systolic blood pressure (SBP), and diastolic blood pressure (DBP), are summarized in Table 1.

### 3.2. Number of Measurements

**Figure 2:**
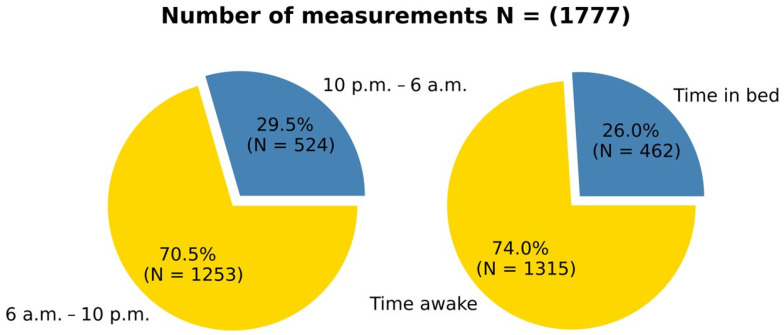
Number of measurements: The figure illustrates the distribution of the blood pressure measurements. The left pie chart represents the distribution of measurements based on the standardized nighttime period between 10 p.m. and 6 a.m. It is divided into two segments labelled ‘10 p.m.–6 a.m.’ and ‘6 a.m.–10 p.m.’. The values inside the segments represent the percentage of and the corresponding absolute number (N) of measurements. The right pie chart shows the distribution of blood pressure measurements based on the time in bed. Like the left chart, the values inside the segments indicate the percentage and absolute number of measurements (N).

Regarding the distribution of BP measurements, we compared the number of measurements obtained during the standardized nighttime period between 10 p.m. and 6 a.m. with those during the TIB. Out of a total of 1777 measurements, 524 (29.5%) measurements took place between 10 p.m. and 6 a.m. During the TIB, BP was measured 462 (26.0%) times (Figure 2).

**Figure 3:**
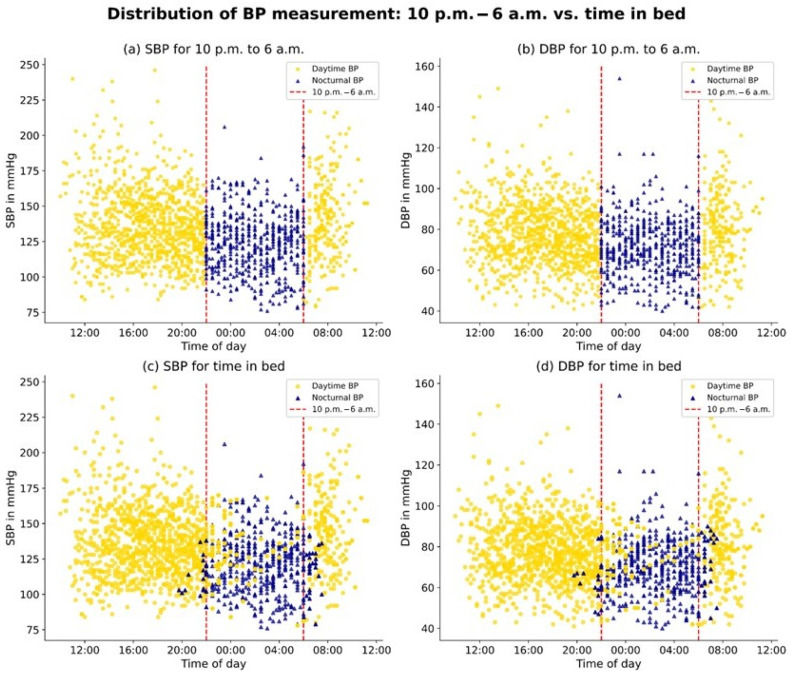
Distribution of BP measurements: 10 p.m.–6 a.m. vs. time in bed.: The figure consists of four panels depicting the relationship between SBP (systolic blood pressure) and DBP (diastolic blood pressure) and time of day. Panels (**a**,**b**) display blood pressure measurements for the standardized nighttime period between 10 p.m. and 6 a.m., while panels (**c**,**d**) show blood pressure measurements for time in bed.

All measurements of the dataset, distinguishing between the time from 10 p.m. to 6 a.m. and the TIB, are illustrated in Figure 3.

### 3.3. Differences in Patient Mean BP

**Figure 4:**
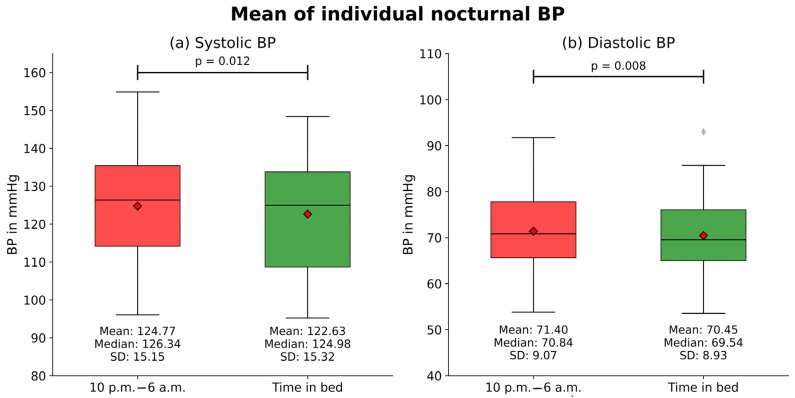
Mean of individual nocturnal BP: This figure compares the patient-specific mean systolic and diastolic blood pressure (BP) during the two periods of time: 10 p.m.–6 a.m. and the time in bed. The boxplots show the patient-specific mean systolic (**a**) and diastolic (**b**) nocturnal blood pressure values of all (N = 30) patients. The p-values represent the results from comparing the groups with the Wilcoxon test for paired non-parametric samples. The small red diamond indicates the mean values.

We observed differences in both the mean values of SBP (*p* = 0.01) and DBP (*p* < 0.01) between the fixed time interval 10 p.m. to 6 a.m. and the TIB. During the period from 10 p.m. to 6 a.m., the average patient mean SBP was 124.8 ± 15.2 mmHg, and the average DBP was 71.4 ± 9.1 mmHg. However, during the TIB, the mean SBP was 122.6 ± 15.3 mmHg, while the mean DBP was 70.5 ± 8.9 mmHg (Figure 4).

**Figure 5:**
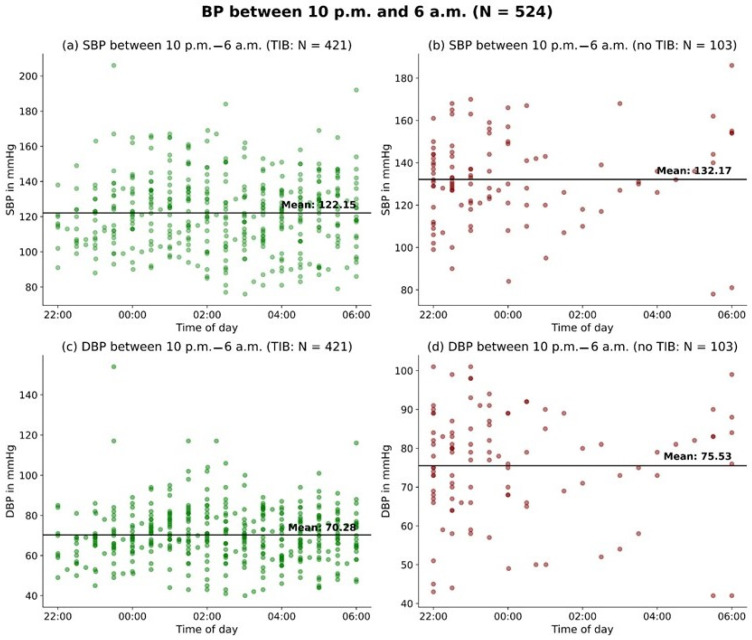
BP during 10 p.m.–6 a.m.: Plots showing blood pressure (BP) during the 10 p.m.–6 a.m. measurement. Each plot displays the mean BP as a solid line. Panel (**a**) shows the systolic BP between 10 p.m.–6 a.m. while also in bed (during TIB). Panel (**b**) depicts the systolic BP between 10 p.m. and 6 a.m. while not in bed (no TIB). Panel (**c**) illustrates the diastolic BP between 10 p.m. and 6 a.m. while also in bed (during TIB). Panel (**d**) displays the diastolic BP between 10 p.m. and 6 a.m. while not in bed (no TIB). TIB = time in bed.

The mean SBP during 10 p.m. to 6 a.m. while patients were also in bed (during TIB) was 122.2 ± 20.6 mmHg. Measurements taken during the same period in which patients were not in bed yielded an average SBP of 132.2 ± 20.5mmHg (*p* < 0.01) (Figure 5).

Similarly, for DBP, the mean value for patients in bed between 10 p.m. and 6 a.m. was 70.3 ± 13.5 mmHg, while the mean value during the same period when patients were not in bed was 75.5 ± 14.1 mmHg (*p* < 0.01) (Figure 5).

### 3.4. Individual Analysis

In relation to the standard nighttime period from 10 p.m. to 6 a.m., we identified 18 (60%) of the subjects as non-dippers, 9 (30%) as dippers, and 3 (10%) as extreme dippers. 

**Figure 6:**
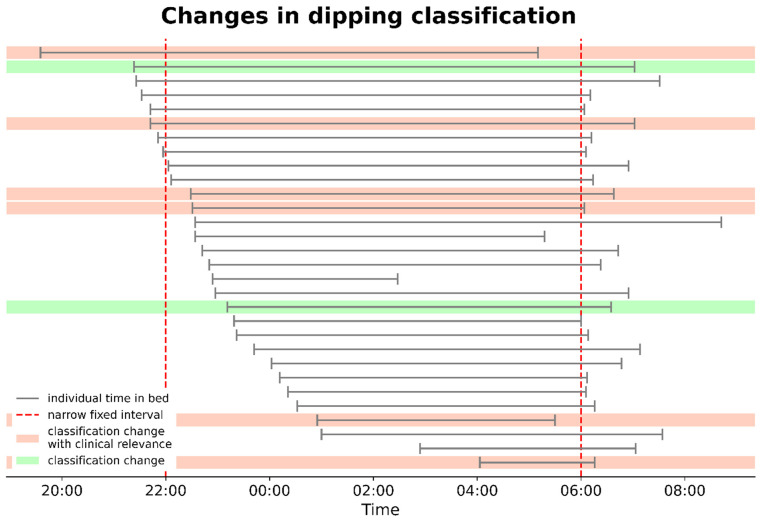
Changes in dipping classification: The figure illustrates the changes in dipping classification based on the time in bed for individual patients. Classification changes are depicted in green, classification changes with clinical relevance (from non-dipper/reverse dipper to dipper/extreme dipper) in red. Each horizontal line represents an individual participant (N = 30), and the vertical segment at the beginning and at the end of each line indicate the start and end of each patients’ time in bed. The red dashed lines indicate the conventional cut-off sleep times (10 p.m.–6 a.m.) of the narrow-fixed interval. The patients are sorted in ascending order based on their time of lying in bed, starting from the earliest.

However, when the BP was assessed based on TIB, altered classifications emerged: 10 (33.3%) as non-dippers, 15 (50%) as dippers, 3 (10%) as extreme dippers, and 2 (6.7%) reverse dippers. In total, eight patients (26.7%) changed their dipping profile, with six (20%) changing from clinically undesirable dipping profiles (non-dipper/reverse dipper) to clinically more beneficial dipping patterns (dipper/extreme dipper) (Figure 6).

### 3.5. Effect of TIB on Dipping Patterns

**Figure 7:**
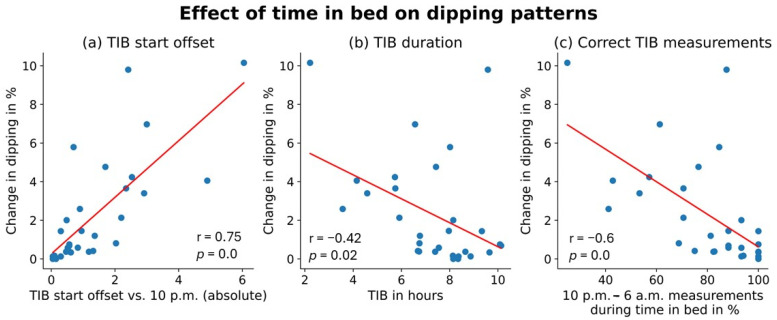
Effect of time in bed on dipping patterns: The figure illustrates the correlation between absolute time in bed offset compared to 10 p.m. (**a**) Total time in bed duration; (**b**) the percentage of measurements from 10 p.m. to 6 a.m. which were also during time in bed; (**c**) with the change in dipping pattern behaviour. TIB = time in bed.

Our data revealed that the absolute offset between the start of the TIB and the beginning of the conventional definition of nighttime (10 p.m.) correlates with the change in dipping behaviour (r = 0.75, *p* < 0.01). Similarly, the change in dipping behaviour between the conventional definition and the TIB is negatively correlated with the total TIB (r = −0.42, *p* = 0.02) and with the percentage of BP measurements taken between 10 p.m. and 6 a.m. which were also taken during TIB (r = −0.60, *p* < 0.01) (Figure 7).

## 4. Discussion

BP assessment, particularly during nocturnal hours, is crucial in evaluating cardiovascular health and predicting the risk of cardiovascular events [2,21]. This pilot study aimed to investigate the comparability between the standardized nighttime period from 10 p.m. to 6 a.m. and the TIB with regard to nocturnal BP and its dipping behaviour. Further, our goal was to evaluate the clinical relevance of these differences for individual patients.

Evidence showing that fixed nighttime intervals are not necessarily aligned with individual TIB is available. Multiple investigations have shown that narrow-fixed nighttime intervals (excluding the hours when individual difference in time to bed and time to get up are more frequent) might be a more robust approach to assessing nocturnal BP patterns than the wide-fixed time intervals (e.g., 8 p.m. to 8 a.m.), but at the price of reducing overall number of available nighttime measurements [22,23,24]. Further studies have emphasized the importance of patient or at least sub-cohort individualized analyses [25,26]. It has been also shown that patient self-reported sleep times (e.g., through a diary) only weakly agrees with TIB derived from actigraphy [14,15,27]. A merit of our study is to have assessed TIB accurately by means of actigraphy and a position sensor, rather than by using patients’ subjective reports.

While the latest guidelines from the European Society of Hypertension recommend a fixed measurement interval of 20 min during day and night [1,28,29], at the time when our data collection started, BP measurements were obtained every half hour between 10 p.m. and 6 a.m., and every 15 min between 6 a.m. and 10 p.m., an approach frequently adopted in daily practice. Our findings reveal large differences between BP values measured during the TIB compared to those obtained during the standard fixed nighttime period. Both SBP and DBP levels were significantly lower during the TIB. These findings indicate that relying solely on the standardized fixed nighttime period may lead to errors in the estimation of cardiovascular risk due to inadequate assessment of the actual, physiological, and clinically relevant nocturnal BP levels.

These differences have important clinical implications: accurate determination of nighttime BP is crucial for appropriately assessing individual cardiovascular risk [2,30]; evaluating nocturnal BP levels based on TIB could provide a more accurate assessment of patient-specific cardiovascular risk. This is particularly important as misjudging nighttime BP can result in suboptimal management and treatment decisions, leading to increased rates of cardiovascular events and associated deaths.

Furthermore, our study revealed large differences in the classification of dipping patterns based on the standardized nighttime period compared to that based on TIB. A notable proportion (20%) of patients were reclassified from non-dipper to dipper when the BP measurements were based on the TIB. A further 6.7% of patients changed from non-dipper to the even more adverse reverse-dipper profile. From a therapeutic viewpoint, non-dippers and reverse dippers might require additional interventions, such as adjusting medication regimens or implementing lifestyle modifications, to improve their nighttime BP control. In contrast, individuals with a dipping profile and normal nighttime BP levels may not require additional therapeutic measures [2,31,32,33]. This highlights the importance of accurately determining the TIB to make precise and correct classifications of the dipping patterns.

We observed a strong correlation of dipping pattern with duration of TIB, absolute offset between the start of TIB and start of the fixed nighttime period (10 p.m.), and number of correct TIB measurements. The findings highlight that accurate assessment of sleep duration and time to fall asleep play a pivotal role in the determination of nocturnal BP and dipping pattern classification.

This has two consequences: On the one hand, the data imply that differences in behaviour (e.g., when a patient is going to bed) can be clinically relevant and should be investigated. On the other hand, when correcting measurements for this behavioural component, the remaining “non-dipping” patterns are likely to depend on pathophysiological mechanisms and therefore might be even better predictors of cardiovascular outcome. This emphasizes the need for a comprehensive assessment nocturnal BP and dipping patterns that may include the concept of TIB. 

It is important to acknowledge the limitations of our study, including the limited sample size of this pilot study and the single centre where data were collected. These factors may restrict the generalizability of our findings and might have exposed our work to potential selection bias. Future studies with larger sample sizes and conducted in diverse clinical settings and patient populations are thus warranted to validate our findings and enhance the external validity of the results. Due to the limited sample size, detailed analyses regarding gender-specific differences, such as nocturnal BP changes in relation to TIB, were not conducted as they would have been greatly underpowered. Further, the TIB is not a perfect measure of true sleep. However, it is questionable whether the additional technical requirements for correctly assessing *true* sleep times are warranted by the unclear additional clinical benefits that such an assessment might carry over the use of the TIB estimated by means of actigraphy and position sensors.

## 5. Conclusions

Our study delivers insights into the assessment of nocturnal BP and emphasizes the importance of accurately determining the TIB. Our findings indicate that the standardized nighttime period from 10 p.m. to 6 a.m. does not adequately represent individual TIB patterns, leading to potential misclassification of cardiovascular risk and dipping patterns. Incorporating the TIB in the evaluation of nocturnal BP can likely provide a more accurate assessment of cardiovascular risk and improve clinical decision making.

In the context of our findings, further research and validation are warranted to support the integration of TIB assessment into clinical practice, ultimately optimizing personalized cardiovascular risk evaluation and patient care.

## Figures and Tables

**Table 1:** Mean values and standard deviation for age, height, weight, body mass index, and systolic and diastolic BP of all patients, by sex.

	Total (N = 30)		Male (N = 21)		Female (N = 9)	
	Mean	SD	Mean	SD	Mean	SD
**Age** **[years]**	64.9	8.8	65.9	6.8	62.7	12.0
**Height** **[cm]**	171.9	7.9	174.3	7.0	166.7	7.3
**Weight** **[kg]**	85.6	13.3	89.4	12.9	77.0	9.7
**BMI** **kg/m^2^**	28.9	3.8	29.4	3.9	27.7	3.3
**SBP** **[mmHg]**	133.5	24.7	135.2	22.9	129.8	28.2
**DBP** **[mmHg]**	76.3	15.7	77.0	15.4	74.5	16.2

BMI = body mass index; SBP = systolic blood pressure; DBP = diastolic blood pressure; SD = standard deviation.

## Data Availability

The original contributions presented in the study are included in the article, further inquiries can be directed to the corresponding author.
